# CLAIRE: Constrained Large Deformation Diffeomorphic Image Registration on Parallel Computing Architectures

**DOI:** 10.21105/joss.03038

**Published:** 2021-05-30

**Authors:** Malte Brunn, Naveen Himthani, George Biros, Miriam Mehl, Andreas Mang

**Affiliations:** 1 Institute for Parallel and Distributed Systems, University Stuttgart; 2 Oden Institute for Computational Engineering and Sciences, The University of Texas at Austin; 3 Department of Mathematics, University of Houston

## Abstract

CLAIRE (Mang & Biros, 2019) is a computational framework for **C**onstrained **LA**rge deformation diffeomorphic **I**mage **RE**gistration (Mang et al., 2019). It supports highly-optimized, parallel computational kernels for (multi-node) CPU (Gholami et al., 2017; Mang et al., 2019; Mang & Biros, 2016) and (multi-node multi-)GPU architectures (Brunn et al., 2020, 2021). CLAIRE uses MPI for distributed-memory parallelism and can be scaled up to thousands of cores (Mang et al., 2019; Mang & Biros, 2016) and GPU devices (Brunn et al., 2020). The multi-GPU implementation uses device direct communication. The computational kernels are interpolation for semi-Lagrangian time integration, and a mixture of high-order finite difference operators and Fast-Fourier-Transforms (FFTs) for differentiation. CLAIRE uses a Newton–Krylov solver for numerical optimization (Mang & Biros, 2015, 2017). It features various schemes for regularization of the control problem (Mang & Biros, 2016) and different similarity measures. CLAIRE implements different preconditioners for the reduced space Hessian (Brunn et al., 2020; Mang et al., 2019) to optimize computational throughput and enable fast convergence. It uses PETSc (Balay et al., n.d.) for scalable and efficient linear algebra operations and solvers and TAO (Balay et al., n.d.; Munson et al., 2015) for numerical optimization. CLAIRE can be downloaded at https://github.com/andreasmang/claire.

## Statement of Need

Image registration is required whenever images are taken at different points in time, from different viewpoints, and/or using different imaging modalities and these images need to be compared, combined, or integrated (Fischer & Modersitzki, 2008; Modersitzki, 2004, 2009; Sotiras et al., 2013). Image registration is an inverse problem. The inputs to this inverse problem are two (or more) images *m*_0_(*x*) (the template image) and *m*_1_(*x*) (the reference image) of the same object. The task of image registration is to find a plausible map *y*(*x*) that establishes spatial correspondences between the reference and template image, i.e., *m*_0_(*x*) ≈ *m*_1_(*y*(*x*)). In CLAIRE the set of admissible spatial transformations *y* is limited to diffeomorphisms, i.e., maps *y* that are continuous, one-to-one, and have a smooth inverse. CLAIRE is related to a prominent class of formulations for these types of problems referred to as large-deformation diffeomorphic metric mapping (Beg et al., 2005; Trouvé, 1998; Younes, 2010).

Diffeomorphic image registration is an indispensable tool in medical image analysis (Sotiras et al., 2013). Computing diffeomorphisms that map one image to another is expensive. Deformable image registration is an infinite-dimensional problem that upon discretization leads to nonlinear optimality systems with millions or even billions of unknowns. For example, registering two typical medical imaging datasets of size 256^3^ necessitates solving for about 50 million unknowns (in our formulation). Additional complications are the ill-posedness and non-linearty of this inverse problem (Fischer & Modersitzki, 2008). Consequently, image registration can take several minutes on multi-core high-end CPUs. Many of the available methods reduce the number of unknowns by using coarser resolutions either through parameterization or by solving the problem on coarser grids; they use simplified algorithms and deliver subpar registration quality. In the age of big data, clinical population studies that require thousands of registrations are incresingly common, and execution times of individual registrations become more critical. We provide technology that allows solving registration problems for clinical datasets in seconds. In addition, we have made available to the public a software that works on multi-node, multi-GPU architectures (Brunn et al., 2020, 2021) that allows the registration of large-scale microscopic imaging data such as CLARITY imaging (Kutten et al., 2017; Tomer et al., 2014).

## Highlights

CLAIRE can be used to register images of 2048^3^ (25 B unknowns) on 64 nodes with 256 GPUs on TACC’s Longhorn system (Brunn et al., 2020). CLAIRE has been used for the registration of high resolution CLARITY imaging data (Brunn et al., 2020). The GPU version of CLAIRE can solve clinically relevant problems (50 M unknowns) in approximately 5 seconds on a single NVIDIA Tesla V100 (Brunn et al., 2020). CLAIRE has also been applied to hundreds of images in brain tumor imaging studies (Bakas et al., 2018; Mang et al., 2017; Scheufele et al., 2021), and has been integrated with models for biophysics inversion (Mang et al., 2018, 2020; Scheufele et al., 2019, 2021; Scheufele, Subramanian, Mang, et al., 2020; Subramanian et al., 2020) and Alzheimer’s disease progression (Scheufele, Subramanian, & Biros, 2020). CLAIRE uses highly optimized computational kernels and effective, state-of-the-art algorithms for time integration and numerical optimization. Our most recent version of CLAIRE features a Python interface to assist users in their applications.

We provide a detailed documentation on how to execute, compile, and install CLAIRE on various systems at our deployment page https://andreasmang.github.io/claire.

## Mathematics

CLAIRE uses an optimal control formulation. The diffeomorphism *y*(*x*) is parameterized using a smooth, stationary velocity field *v*(*x*). Given the template image *m*_0_(*x*) and the reference image *m*_1_(*x*), this velocity is found by solving the partial-differential equation constrained optimization problem of the form

$${\text{minimize}}_{v,m}\text{ dist}\left(m\left(x,t=1\right),m_{1}\right)+\alpha \text{ reg}\left(v\right)$$

subject to

$$\partial_{t}m\left(x,t\right)+v\left(x\right)\cdot \nabla m\left(x,t\right)=0\;m\left(x,t=0\right)=m_{0}\left(x\right)$$

The first term in the objective functional measures the proximity of the deformed template image *m*(*x, t* = 1) and the reference image *m*_1_(*x*). The default option availble in CLAIRE is an *L*^2^-distance. The second term controls the regularity of *v*. CLAIRE features different Sobolev norms. The default option is an *H*^1^-seminorm. The constraint models the deformation the template image (i.e., the transport of the intensities of *m*_0_(*x*)). CLAIRE also features additional hard constraints for controlling the divergence of *v*(*x*) (Mang & Biros, 2016). For optimization, we use the method of Lagrange multipliers and solve the associated Karush–Kuhn–Tucker optimality system using a Newton–Krylov reduced space method (Mang & Biros, 2015, 2015).
